# Worldviews of science teachers in educational-technological context as a key factor in digitalization of teaching practices

**DOI:** 10.12688/f1000research.28074.1

**Published:** 2021-02-04

**Authors:** Dina Tsybulsky, Yulia Muchnik-Rozanov

**Affiliations:** 1 Education in Science and Technology, Technion - Israel Institute of Technology, Haifa, Israel

**Keywords:** Educational-technological context, school digitalization, teachers’ worldviews, teachers’ beliefs, linguistic analysis

## Abstract

**Background:** This research deals with science teachers' worldviews in the educational-technological context. Obtaining a deeper insight into teachers' discourse regarding school digitalization and understanding teachers' worldviews in the educational-technological context may be viewed as crucially important since the latter tends to play a central role in the process of digitalization of teaching practices.

**Methods:** This study addresses the following questions: (1) Was there a difference between the teachers regarding their foci of attention expressed via personal pronouns? (2) Was there a difference between the teachers in terms of the quality and degree of their emotional immersion in the discussed topic expressed through the use of emotion words? (3) What are the semantic fields of the word clusters that include the lexemes technology and digital, and do they implicitly convey differences in teachers' understanding of school digitalization? The data were extracted by means of in-depth interviews with 38 Israeli science teachers. The linguistic analysis was employed to examine teachers' language behavior.

**Results:** The results point out the differences in teachers' worldviews, manifested through language behavior. In particular, the differences between the three groups of teachers (outside observers, circumspect participants, and conscientious participants) were found regarding their foci of attention, the level of emotional immersion, and their implicitly conveyed understanding of the digitalization of teaching practices.

**Conclusions:** The teachers' worldviews are the key element for understanding what it means to be or not to be a teacher in a digital society. In addition, our study demonstrates that linguistic analysis in educational research is a promising methodological approach that can render an in-depth and comprehensive picture of the explored phenomenon.

## Introduction

The digital age has brought profound changes in the global society. On the one hand, it made our lives easier, added new possibilities for humans while impacting human society ( Ganascia, 2015). On the other hand, such transformation is naturally accompanied by challenges. Among the challenges that these enhancements inevitably created is the blurring of boundaries between personal and public ( Oates, 2015), which implicate work-life balance, relationships, connections, and social interactions. Floridi (2015) describes the digital age as the era of hyper-connectivity, which adds another challenge to people who are connected to multiple spaces (online and offline) at the same time.

Challenges that concern technology integration ( Guzey & Roehrig, 2009), confidence in ICT literacy ( Hsu *et al*., 2012), content knowledge ( Schneider & Plasman, 2011), instruction ( Herro *et al*., 2019), and pedagogical beliefs ( Margot & Kettler, 2019) of teachers are well addressed in the literature ( Davis *et al*., 2006). These and other challenges, as well as clear advantages brought by the digital age, tend to affect teachers’ worldviews regarding the process of digitalization in the educational-technological context. Obtaining a more in-depth insight into teachers’ discourse regarding school digitalization and understanding teachers’ worldviews in the educational-technological context may be viewed as crucially important since the latter tends to play a central role in the process of digitalization of teaching practices.

### Theoretical background

***Teachers’ worldviews.*** Worldviews – collections of beliefs relating to various aspects of our human experience – are often viewed as the foundation that influences the way we think about and responds to reality ( deWitt, 2018; Hiebert, 2008; Naugle, 2002). Our worldview determines our fundamental ideas about the world, how we analyze our surroundings, and the actions we take.

Lately, the emergence of a digital society has been viewed as a change in people’s worldview ( Ess, 2015; Floridi, 2014). Recently conducted studies in the educational context suggest that teachers’ worldviews are transformed in response to the profound change in the global society ( Tsybulsky & Levin, 2017; Tsybulsky & Levin, 2019). Using content and structural analyses, the study of Tsybulsky and Levin (2019) found that science teachers’ worldviews could be classified into the following categories:

1. **The Outside Observer**: These teachers are aware of the changes that occurred in the global society, yet they observed these phenomena from the outside. They are not consciously involved in the transformation accompanying the transition to a digital society. Only minor changes in their worldviews were observed.
2. **The Circumspect Participant**: These teachers are aware of the digital transformation taking place in the global society. They are consciously involved in these changes but do not always support them either cognitively or emotionally. Apparent changes in their worldviews were observed.
3. **The Conscientious Participant**: Are not only these teachers aware of the global developments, but they are also knowingly involved in making these changes take place. Their worldviews have been significantly transformed in response to school digitalization.

Following the above study ( Tsybulsky & Levin, 2019), the present work seeks to explore science teachers’ worldviews in the educational-technological context by observing their language behavior. We expected that the variations between the worldviews of teachers of the above-mentioned categories would be manifested in their language and, thus, could be revealed by analyzing the distinctive linguistic markers. To the best of our knowledge, only a few studies have addressed educational phenomena by exploring language behavior ( Tsybulsky & Muchnik-Rozanov, 2019; Ursúa & Vasquez, 2008). However, our previous studies suggest that linguistic analysis is a promising method for understanding implicitly conveyed messages (in this context, implicitly conveyed messages in teachers’ narratives) (e.g., Muchnik-Rozanov & Tsybulsky, 2019; Muchnik-Rozanov & Tsybulsky, 2020).

### Linguistic analysis

The present study focuses on the theoretical frameworks and approaches addressed below.

Firstly, Systemic Functional Linguistic (SFL) approach proposed by Halliday (1978) views language as a system of grammatical and lexical choices that a language user makes to convey certain meanings and thereby function in society ( Halliday *et al*., 2014). In the process of communication, the focal point of the speakers’ attention can be placed on the external settings as well as on the interlocutors’ deep feelings (e.g., Pennebaker, 2011). Our study follows a beaten track within the SFL framework by analyzing the referential system of language (e.g., Fine, 2006; Rude *et al*., 2004; Smirnova *et al*., 2015). Specifically, this work examines how the participants’ foci of attention are realized through their choices of references (personal pronouns).

Secondly, Tausczik & Pennebaker (2010), as well as Holmes *et al*. (2007), propose to analyze speakers’ or writers’ emotion words to understand the extent to which they are emotionally involved in the discussion on a specific topic. In addition, the analysis of the valence of emotion words pertains to understanding how the participants perceive various phenomena (e.g., ‘glad,’ ‘happy,’ ‘moving’ refer to positively perceived phenomena, whereas ‘cruel,’ ‘hard,’ ‘neglected’ are used to describe phenomena perceived as negative). In this study, emotion words were analyzed to explore the extent to which the participants are emotionally involved in the discourse in the educational-technological context.

The third linguistic theoretical framework of this study is based on Semantic Theory and focuses on the analysis of semantic fields, where a semantic field is defined as a set of interrelated lexemes that cover a particular aspect of reality ( Brinton, 2000). The analysis of semantic fields can be employed to understand the worldviews among various communities as well as individuals. In the present study, the semantic fields of specific word sequences were analyzed to extract participants’ covertly expressed understanding of school digitalization.

***Linguistic analysis in education.*** Despite its wide utility in psychology (e.g., Freda *et al*., 2015; Weintraub, 1989) and psychiatry (e.g., Bersudsky *et al*., 2005; Fine, 2006; Nienow & Docherty, 2004; Smirnova *et al*., 2015), linguistic analysis has been relatively seldom employed in educational research. For example, linguistic analysis has been used to explore the notion of leadership in the context of educational administration ( Anderson & Mungal, 2015; Crowhurst & Emslie, 2018). Another instance of applying linguistic analysis to educational research is researching interventions for children with disabilities in the context of special education ( Imms *et al*., 2016). In addition, linguistic analysis of textbooks has been found to contribute to multicultural educational research by offering an insight into the role of textbooks in helping students find their own voice ( Curdt-Christiansen & Weninger, 2015).

A few studies have been dedicated to the analysis of associations between teaching, learning, and students’ identities across time and context ( Tamatea *et al*., 2008; Sfard & Prusak, 2005) teachers’ ideological perspectives on their work ( Llewellyn, 2005); inservice teachers’ identity ( Heyd-Metzuyanim, 2019; Heyd-Metzuyanim & Shabtay, 2019), and preservice teachers’ identity ( Tsybulsky & Muchnik-Rozanov, 2019; Ursúa & Vasquez, 2008).

In this study, a linguistic analysis was employed in educational research to explore inservice teachers’ worldviews in the educational-technological context, as expressed in their language behavior. It should be noted that, to the best of our knowledge, existing studies in the field have not utilized linguistic analysis to explore teachers’ worldviews in the realm of education. 

## Methods

### The research questions

The present research addressed the following questions:

1. Was there a difference between the teachers in terms of their foci of attention expressed via personal pronouns?
2. Was there a difference between the teachers in terms of the quality and degree of their emotional immersion in the discussed topic expressed through emotion words?
3. What are the semantic fields of the word clusters that include the lexemes *technology* and *digital,* and do they implicitly convey differences in teachers’ understanding of school digitalization?

### The context of the study

This study’s participants were inservice high-school science teachers ( *n* = 38) from a central metropolitan area of Israel. The participants were chosen with several concepts in mind. First, as the previous study aimed to identify teachers’ worldviews in the context of digitalization of teaching practices, science teachers constituted a group that was likely to be more open to and aware of technological developments. This led to the second assumption, namely, that a shift in the predominant sociocultural worldview would be detected first and foremost among those who teach a discipline that is continuously affected by technological progress. Third, students and teachers working and living in the central metropolitan area were likely to be the first to experience such a change, as they represent a high socioeconomic class compared to other parts of the country.

We used a voluntary group, which involved only those teachers who agreed to participate in the study. The participants were recruited by using a mailing list of the National Center of Science Teachers as well as by utilizing professional social networks. The teachers were informed about the research goals and procedure and indicated their willingness to participate by completing a written informed consent form (0% of dropout rate). The study was approved by the Behavioural Sciences Research Ethics Committee of the Technion (Approval number 2018-075).

### Data collection

The data collection tool selected for the study was the in-depth interview. The interviews took place at venues chosen by the interviewees, such as their schools, within the time slots the participants found convenient. Each interview lasted approximately 90 minutes. The focus of the interview protocol ( *Extended data:* Appendix 1 ( Tsybulsky, 2021)) was on participants’ feelings, thoughts, and emotions regarding digital technologies, as these were manifested in their personal and professional lives. The interviews were conducted by DT of this study. She is an expert in educational research, and in qualitative research in particular, with a PH.D. Degree in Science Education. All the interview data were audio-recorded and then transcribed in conventional orthographic local language by a Ph.D. student in science education, a native speaker, who is experienced in transcribing language data.

### Data analysis

As it has been mentioned above (see *Theoretical background*), our analysis focuses on the three groups of teachers defined by the content structural analysis conducted in the earlier study ( Tsybulsky & Levin, 2019): five teachers were assigned to the category of outside observers, 12 were assigned to the category of circumspect participants, and 21 teachers were assigned to the category of conscientious participants.

The linguistic analysis of the data focused on three markers: (1) personal pronouns as the indicators of the foci of attention; (2) emotion words as the indicators of teachers’ emotional involvement in the discussed topic; (3) semantic fields of the words that clustered with the lexemes *technology* and *digital*, as the indicators of participants’ implicit perceptions of school digitalization.

To explore teachers’ **foci of attention** (RQ1), we scrutinized the personal pronouns used by the participants. In an attempt to differentiate between the focus of attention placed on the external world versus inner feelings, the distinction between speech-role (SR) and non-speech-role (NSR) must be made ( Halliday *et al*., 2014; Rochester & Martin, 1977). SR refers to the interlocutors (personal references of the first and the second person), whereas NSR refers to the external settings: places, people, and objects referred to by the speakers (third-person personal references).

Based on Levenston (1970), we used a list of the personal pronouns mentioned above to code all personal references in the transcripts and then calculated the frequency of each type of pronoun. Then the frequencies of first- and second-person references were merged into the SR category. Similarly, the frequencies of all third-person references were merged into the NSR category. We converted the frequency totals into a rate per 1,000 words to control the length of the elicited and transcribed speech samples. The rates for SR references were compared to NSR references in the three groups of teachers: outside observers, circumspect participants, and conscientious participants.

To examine the degree and quality of participants’ emotional **immersion into a topic discussed** during the interview (RQ2), we analyzed two aspects of the emotion words employed by the participants. We considered the positive versus negative valence of the emotion words they employed, which indicate the speakers’ perceptions of their surrounding world, and also calculated the rate at which emotion words were used in one’s language performance has been associated with increased immersion in a described process or event ( Holmes *et al*., 2007; Tausczik & Pennebaker, 2010). To this end, based on Tausczik & Pennebaker (2010), the lexemes and stems listed under the category of *emotion words* in the Linguistic Inquiry and Word Count (LIWC) software dictionary were translated into conventional Hebrew by a native speaker with a PH.D. Degree in Semitic Languages, who is also fluent in English. The transcripts were coded for all the lexemes and word stems in the list, and their frequencies were calculated. The frequency totals for emotion words of positive and negative valence were converted into rate per 1,000 words to control for the length of the elicited and transcribed speech samples. The rate of emotion words was compared among the three groups of teachers: outside observers, circumspect participants, and conscientious participants. In addition, we compared the frequencies of positive and negative emotion words in each of the three groups.

To explore participants’ **implicitly conveyed understanding** of school digitalization (RQ3), we looked for possible variations in the teachers’ word choices when discussing digital-age-related experiences in the context of their everyday professional teaching practices. To this end, the transcripts were coded for all the word clusters containing the two lexemes *digital* and *technology*. Word clusters with one of the required lexemes that, in addition, featured only prepositions, pronouns, and/or auxiliary and modal verbs were omitted from the semantic field analysis. Both the nature of the word clusters containing both the lexemes *digital* and *technology* and their distribution across semantic fields were compared across the three groups of teachers. The semantic distribution was measured as a percentage of the total number of word clusters in each teacher group. Overall, 95,409 words were analyzed throughout 38 transcribed interviews. Data analysis was conducted by YM. She is an expert in linguistic analysis with a PH.D. Degree in Linguistics. 30% of the data were also analyzed by DT using a coding protocol. Inter-coder reliability of 90% was achieved. 

Distinctive linguistic markers, subcategories, coding methods, and examples are summarised in Table 1.

**Table 1.** Distinctive linguistic markers, subcategories, coding method, and examples.

Distinctive Linguistic Markers	Subcategory	Coding Method	Examples
**Personal pronouns** **to examine the focus** **of attention (RQ1)**	Speech-role personal pronouns used by the speakers to refer to the conversation participants	First-person singular ( *I, me, my,* *mine*)	‘ I believe that school must be school’. (Ofer) ‘It takes up most of my time’. (Shiran)
		Personal pronoun of the first- person plural ( *we*, *us our, ours*)	‘There are friends who have slipped away, and we get in touch from time to time’. (Leilah)
		Personal pronoun of the second person ( *you, your*, *yours*)	‘ You cannot play with your phone because you are sitting in a lesson’. (Rose)
	Non-speech-role personal pronouns used by the speakers to refer to the situational context of the conversation (places, people, or objects)	Personal pronouns of the third- person singular ( *he, his, him,* *she, her, hers, it, its*)	‘ He (a virtual friend) is not your true friend’. (Rinat) ‘I used to have a student..., and she asked for permission to leave the class five minutes earlier’. (Lailah) ‘The level of teaching is not what people think it is’. (Ofer)
		Personal pronouns of the third- person plural ( *they, them, their, theirs*)	‘Today, there is a new application to do the same, and they are very similar’. (Shani)
**Emotion words** **to measure the** **quality and level** **of immersion in** **the discussed** **phenomenon or** **process (RQ2)**	Positive	The lexemes and stems listed under the category’ positive emotion words’ in the LIWC software dictionary (e.g. nice, confident, sincere, etc.)	‘It’s very nice’. (Tamar) ‘He is like a friend of yours’. (Rinat) ‘This is really advanced technology’. (Ohad) ‘Technology serves me both at work and in my personal life’. (Michal)
	Negative	The lexemes and stems listed under the category ’negative emotion words’ in the LIWC software dictionary (e.g., hurt, ugly, nasty)	‘It’s difficult’. ‘The application is very primitive’. (Ohad) ‘You can use technology even for doing silly things’. (Neta)
**Semantic fields to** **study the teachers’** **understanding of** **school digitalization** **(RQ3)**	Semantic field of pedagogical practices and learning process	The lexemes that co-occur with *digital* and *technology* to denote teaching-learning or school practices	*to screen, to lead, process, teach, stimuli,* *laboratory, calculator, frontal, write, class,* *learning, experiential, school bag,* etc.
	Semantic field of tools and means	The lexemes that co-occur with *digital* and *technology* to denote technology to enhance teaching	*messages, gadgets, devices, means, data,* *sites, technological platform, animation,* *simulation, imaging, tablet, equipped,* *beepers, analogic,* etc.
	Semantic field of emotional states	The lexemes that co-occur with *digital* and *technology* to denote emotions associated with the phenomenon of digitalization of education	*happy, scary, forbidden, disappointing*, etc.
	Semantic field of rapid development	The lexemes that co-occur with *digital* and *technology* to denote unprecedented development of digital technology	*lead, development, jumped, exponentially,* *push*, etc.
	Semantic field of youth/young generation	The lexemes that co-occur with *digital* and *technology* to denote the existence of a new generation of pupils and students	*the young, children, were born, new* *generation,* etc.

## Results

### The foci of attention revealed through the use of personal pronouns (RQ1)

It was found that there are some differences in the foci of attention between the three groups of teachers. Since the conscientious participants were predominantly focused on the situational context, they tended to use more non-speech-role references than the outside observers or the circumspect participants (57 vs. 39 and 45, respectively). These findings point out that those teachers who are consciously involved in the digital transformation of the global society refer to the external world more frequently than do other groups of teachers. The circumspect participants used fewer speech-role references than the outside observers or the conscientious participants (90 vs. 100 and 95). Such language behavior tends to signify a lack of personal involvement in the changes accompanying the process of digitalization. In this group and the outside observers, similar rates of non-speech-role references were found (45 and 39, respectively). These findings indicate that those teachers did not show more interest in the external world than outside observers, who felt alienated from the transition to a digital society.

Regarding SR references, it was found that the outside observers used slightly more of this reference type than either the circumspect or the conscientious participants (100 vs. 90 and 95, respectively). The teachers in this group also used fewer NSR references than the circumspect participants or the conscientious participants (39 vs. 45 and 57, respectively). The above findings indicate that the outside observers were more focused on their own experiences and feelings than two other groups of teachers while discussing the transformations accompanying society’s digitalization in general and the digitalization of school in particular. Table 2 presents a summary of the findings regarding RQ1.

**Table 2.** Summary of the findings regarding the use of personal pronouns as indicating teachers’ foci of attention.

Measured Variable	Distinctive Linguistic Markers	Outside Observers	Circumspect Participants	Conscientious Participants
		Frequencies (rates per 1,000 words)		
The foci of attention	Speech-Role References	1,239 (100)	2,826 (90)	4,943 (95)
	Non-Speech-Role References	483 (39)	1,413 (45)	2,965 (57)
**Total Words**		**12,319**	**31,326**	**51,764**

### The degree of immersion in a discussed topic demonstrated through emotion words (RQ2)

It was found that all teachers used emotion words (both positive and negative). However, there were some differences in the frequency rates of emotion words between the three observed groups. Firstly, our findings show that the conscientious participant group used the fewest number of emotion words compared to the teachers in the outside observer and circumspect participant groups (19 vs. 30 and 17). These findings suggest that the conscientious participant group was less immersed in the discussion on digitalization in an educational context than the teachers who were defined as the outside observers but more immersed than the teachers assigned to the circumspect participant group. Secondly, it was found that the language of the outside observers indicated that this group was more immersed in the phenomenon of a digital society than either of the other two groups (30), a finding that—although surprising— may be traced to their firm belief that the changes accompanying school digitalization are superficial and, perhaps, to a desire to conceal their real sense of alienation. Table 3 presents the summary of the findings regarding RQ2.

**Table 3.** Summary of the findings regarding the use of emotion words as indicating the level of teachers’ emotional immersion into a discussed topic.

Measured Variable	Distinctive Linguistic Markers	Outside Observers	Circumspect Participants	Conscientious Participants
		Frequencies (rates per 1,000 words)		
The level of immersion into a discussed topic	Positive Emotion Words	245 (20)	335 (11)	586 (12)
	Negative Emotion Words	124 (10)	206 (7)	376 (7)
	Total Emotion Words	369 (30)	541 (17)	962 (19)
**Total Words**		**12,319**	**31,326**	**51,764**

In regard to the analysis of negative and positive emotion words as two separate coding categories, our findings demonstrate the lowest rate of negative emotion words used by the teachers in the circumspect participant group (7). This finding reflects the teachers’ awareness of school digitalization as a necessary and inevitable process that, nevertheless, is accompanied by negative emotions. These negative emotions are associated with feeling external pressure to adjust to the digitalization process despite both affective and cognitive alienation.

### The differences between the three groups of teachers revealed through the semantic field analysis (RQ3)

Our findings show that five semantic fields were associated with the lexemes *digital* and *technology:* pedagogical practices and the learning process, means and tools, emotional states, the rapid development of technology, and the younger generation. The semantic field of *pedagogical practices and the learning process* included such words as ‘teacher,’ ‘lesson,’ ‘understand,’ ‘learners,’ ‘struggling students,’ etc. The semantic field of *means and tools* referred to the words like ‘presentation,’ ‘virtual lab,’ ‘WhatsApp group,’ ‘computer,’ etc. The semantic field of *emotional states* comprised the words like ‘feel,’ ‘upset,’ ‘glad,’ ‘scared,’ etc. The semantic field of the *rapid development of technology* was associated with such words as ‘changes,’ ‘development,’ ‘progress,’ ‘technology,’ ‘ICT,’ etc. Finally, the semantic field of *the younger generation* incorporates words like ‘different generation,’ ‘future generation,’ *‘*digital natives,’ ‘young people,’ ‘children,’ etc.

We found some differences between the three groups of teachers regarding the distribution across the semantic fields. The following semantic fields were observed among the conscientious participants: 1) pedagogical practices and the learning process; 2) means and tools; 3) emotional states. For this group of teachers, the most saturated semantic field was associated with *pedagogical practices and the learning process* (78%). By contrast, a significantly smaller number of clusters were produced by that group referred to the semantic field of *means and tools* (11%) and *emotional states* (6%). In addition, 5% of the clustering lexemes belonged to various semantic fields that could not be grouped.

In the circumspect participant group, the findings revealed the same three semantic fields (pedagogical practices and the learning process; means and tools; and emotional states), but a different distribution was observed (29%, 58%, and 7%, respectively.) A total of 6% of the words clustered with *digital* and *technology* belonged to various semantic fields and could not be categorized and analyzed in the present study.

In the outside observer group, we found the associations with all five semantic fields: *emotional states* (34%), *rapid development* (32%), *the younger generation* (18%), *means and tools* (8%), *and pedagogical practices and the learning process* (6%). Throughout the interviews with these teachers, only 2% of the clustering lexemes referred to diverse semantic fields that could not be grouped. Table 4 presents a summary of the findings regarding RQ3.

**Table 4.** Summary of the findings regarding the semantic fields as indicating teachers’ perceptions of school digitalization.

Groups of Teachers	Outside Observers *	Circumspect Participants **	Conscientious Participants ***
**Semantic Fields**			
Pedagogical practices and learning processes	6	29	78
Means and tools	8	58	11
Emotional states	34	7	6
Youth/younger generation	18	0	0
The rapid development of technology	32	0	0
Free lexemes	2	6	5

* The total number of clusters with the lexemes *digital* and *technology–*88 (100%)

** The total number of clusters with the lexemes *digital* and *technology–*311 (100%)

*** The total number of clusters with the lexemes *digital* and *technology–*344 (100%)

These findings reflect the teachers’ implicitly conveyed understanding of school digitalization in the context of their everyday professional teaching practices. The teachers’ vision ranges from total acceptance and successful implementation to hostility and disagreement. High saturation of the semantic field associated with *pedagogical practices and the learning process* shows that the participants view digital technology as an integral part of their classroom experiences and perceive the digitalization process as natural and positive. The high saturation of the semantic field associated with *emotional states* indicates the teachers’ strong emotional involvement. Numerous clusters related to the semantic fields of the *rapid development of technology* and *the younger generation* (semantic fields that were found unique for the outside observer group) point at their awareness of the school digitalization process as well as understanding that the changes brought in by this process are inevitable but not necessarily beneficial for the new generation of students. The high saturation of the *means and tools* semantic field suggests that these teachers perceive the process of school digitalization only at the instrumental and practical level. At the same time, a few clusters associated with the field of *means and tools* reflect a deeper understanding of the digitalization process as a new reality that leads to redefining school practices. 

## Discussion

Teachers reconstruct their identities and worldviews in the digital age, and these changes are conceptual ( Avidov-Ungar & Forkosh-Baruch, 2018; Tsybulsky & Levin, 2019). Throughout this worldview reconstruction, teachers may be seen as belonging to one of three groups, based on their worldviews regarding school digitalization: the outside observer, the circumspect participant, or the conscientious participant ( Tsybulsky & Levin, 2019). This study demonstrates that the worldview reconstruction is so profound that it manifests itself in the teachers’ language behavior.

The previous studies in the field employed a qualitative approach and the content analysis method. While content analysis deals with explicitly conveyed messages and views, the linguistic analysis performed in the current study aims to provide a deeper understanding of the teachers’ worldviews based on implicit aspects that are not related to the content of the conveyed message but rather to the way it is conveyed in terms of language use. In particular, our analysis sought to identify the foci of attention, the quality and degree of emotional immersion, and semantic fields of the word clusters containing *the lexemes digital* and *technology* as manifested in the language found in the transcripts of all three groups of teachers.

The results indicate that the conscientious participants, who are both affectively and cognitively involved in the school digitalization process, were more focused on and interested in the external world than the two other groups of teachers. The teachers in this group exhibited an intermediate level of emotional immersion. These findings indicate that teachers in this group perceived digital social and personal experiences, interactions, and artifacts, as well as the concomitant changes in the surrounding world, as an integral part of their daily lives and, consequently, as an intrinsic constituent of their personal and professional identities.

The observed distribution of semantic fields associated with word clusters containing the lexemes *digital* and *technology* indicates that the teachers who conscientiously participate in school digitalization viewed technology as an inseparable and integral part of this process. Expressions of emotion seldom accompanied descriptions of their digital pedagogical practices, and negative emotions were noticeably rare.

The circumspect participants were less focused on themselves than the two other groups of teachers, suggesting a more substantial differentiation between their personal and professional identities. They showed a relatively low level of emotional involvement when discussing the digitalization of society. As professionals, they were aware of the changes taking place in their realm, and they were willing to be involved in these changes to remain relevant and effective teachers. However, on a personal level, they did not consider themselves to be a part of a digital society and were emotionally distanced from it, which may be related to the paucity of experience in and interactions with the digital habitat. Besides, the findings regarding the semantic fields show that the teachers in this group perceive the process of school digitalization only at the instrumental and practical level. These teachers frequently claimed that ‘Technology is solely a tool.’ What matters is pedagogy’.

More than the two other groups of teachers, the outside observers were focused on themselves and less focused on the surrounding world. They also showed the highest level of emotional immersion. As they explicitly reported their awareness of the transformations taking place around them, these teachers’ language behavior reflects their emotional estrangement and distancing from a digital society (the world in which their students are immersed). The present linguistic analysis indicates that these teachers felt a contradiction between their values and what they observed regarding the transition to a digital society. Teachers’ language behavior in this group reflects their concerns about digital technology and its consequences for teaching and learning. Their worldview regarding ICT development and the accompanying transformations in the global society were manifested through powerful and mostly negative emotions.

In sum, the present study identified differences in the language of the three groups of teachers in terms of the interviewees’ foci of attention, the degree of their emotional immersion when discussing the digitalization of school, and the semantic fields evoked through word clusters containing the lexemes *digital* and *technology*. Moreover, findings of the current linguistic analysis made it possible to delve beneath the surface of explicit statements to reveal more than the teachers’ worldview, namely, their feelings, emotions, concerns, and attitudes. These results corroborate other studies, albeit limited in number, emphasizing the value of linguistic analysis for examining teachers’ perceptions and beliefs ( Llewellyn, 2005; Muchnik-Rozanov & Tsybulsky, 2019; Muchnik-Rozanov & Tsybulsky, 2020; Tsybulsky & Muchnik-Rozanov, 2019).

Although the number of the analyzed words was relatively large in linguistic analysis terms, it may be problematic because it features a certain degree of homogeneity in the selected population. Nonetheless, the methodological framework used herein, specifically examining worldviews by exploring three distinctive linguistic markers (personal pronouns, emotional words, semantic fields of specific word clusters), proved effective. Hence, it is recommended for use in future educational research conducted in a broader context as a way to shed additional light on inservice and preservice teachers’ values and beliefs.

The current study’s contribution is twofold. Firstly, our study contributes to the discourse on school digitalization. The teachers’ worldviews are the key element for understanding what it means to be or not to be a teacher in a digital society. Secondly, our study demonstrates the value of linguistic analysis in the realm of educational research. We believe that linguistic analysis in educational research is a highly promising methodological approach that can render a deep and comprehensive picture of the explored phenomenon.

## Data availability

### Underlying data

The transcripts underlying the results cannot be shared for the following reasons. Firstly, to meet the requirements of the Behavioural Sciences Research Ethics Committee of the Technion, researchers are forbidden to share recorded and/or transcribed interviews with anybody except the research team. An exception is made for publishing findings where anonymous interview quotes can be used. Second, the participants’ anonymity and data protection were insured in the written consent form signed by the participants prior to the commencement of the study. For interested researchers, please contact the Behavioral Sciences Research Ethics Committee of the Technion for access to the data ( bs.ethics.technion@gmail.com). 

### Extended data

DANS: Appendix 1. Interview protocol, https://doi.org/10.17026/dans-27e-pmdu ( Tsybulsky, 2021).

This project contains the following extended data:

- Appendix 1. Interview Protocol

Data are under a DANS license (Open Access for Registered Users), which allows unrestricted access to the data, while stipulating that the user must comply with the Netherlands Code of Conduct for Research Integrity, the General Data Protection Regulation (GDPR) and other applicable laws and regulations.
