# Supplementary material for: Worldviews of science teachers in educational-technological context as a key factor in digitalization of teaching practices
Source: F1000Res. 2021 Jun 16;10:ISF-71. Originally published 2021 Feb 4. [Version 3] doi: 10.12688/f1000research.28074.3 (PMC8240596; doi:10.12688/f1000research.28074.3)
Supplement: Peer review history

# Peer review: Reviewer response for version 3

**DOI:** 10.5256/f1000research.57715.r87700

**Published:** 2021-06-28

**Authors:** Deniz Sarıbaş

**Affiliations:** 1 Elementary Education Department, Istanbul Aydin University, Istanbul, Turkey

This version of the article seems much better. I have nothing to add more. I think the contribution of the article is clearer now.

Is the work clearly and accurately presented and does it cite the current literature?

Yes

If applicable, is the statistical analysis and its interpretation appropriate?

Not applicable

Are all the source data underlying the results available to ensure full reproducibility?

Partly

Is the study design appropriate and is the work technically sound?

Yes

Are the conclusions drawn adequately supported by the results?

Yes

Are sufficient details of methods and analysis provided to allow replication by others?

Partly

Reviewer Expertise:

Science education

I confirm that I have read this submission and believe that I have an appropriate level of expertise to confirm that it is of an acceptable scientific standard.

# Peer review: Reviewer response for version 3

**DOI:** 10.5256/f1000research.57715.r87699

**Published:** 2021-06-16

**Authors:** Noemi Waight

**Affiliations:** 1 Department of Learning and Instruction, University at Buffalo, Buffalo, NY, USA

None. I provided my feedback in the last form.

Is the work clearly and accurately presented and does it cite the current literature?

Yes

If applicable, is the statistical analysis and its interpretation appropriate?

Not applicable

Are all the source data underlying the results available to ensure full reproducibility?

No source data required

Is the study design appropriate and is the work technically sound?

Yes

Are the conclusions drawn adequately supported by the results?

Yes

Are sufficient details of methods and analysis provided to allow replication by others?

Yes

Reviewer Expertise:

Science education; technology design and implementation; nature of technology; teachers' practices with technological-supported, inquiry-based practices.

I confirm that I have read this submission and believe that I have an appropriate level of expertise to confirm that it is of an acceptable scientific standard.

# Peer review: Reviewer response for version 2

**DOI:** 10.5256/f1000research.56745.r85858

**Published:** 2021-06-07

**Authors:** Noemi Waight

**Affiliations:** 1 Department of Learning and Instruction, University at Buffalo, Buffalo, NY, USA

This version of the manuscript was significantly improved and overall, I was satisfied with the author's responses to the feedback. This version of the manuscript was clearly articulated and importantly, the manuscript explicitly addresses the linkage of world views, language usage and how this informs the profiles of teachers. Given this clarity, I now understand the important contributions of this study.

Is the work clearly and accurately presented and does it cite the current literature?

Yes

If applicable, is the statistical analysis and its interpretation appropriate?

Not applicable

Are all the source data underlying the results available to ensure full reproducibility?

No source data required

Is the study design appropriate and is the work technically sound?

Yes

Are the conclusions drawn adequately supported by the results?

Yes

Are sufficient details of methods and analysis provided to allow replication by others?

Yes

Reviewer Expertise:

Science education; technology design and implementation; nature of technology; teachers' practices with technological-supported, inquiry-based practices.

I confirm that I have read this submission and believe that I have an appropriate level of expertise to confirm that it is of an acceptable scientific standard.

# Response

**Published:** 2021-06-10

**Authors:** Dina Tsybulsky

**Affiliations:** Technion - Israel Institute of Technology, Israel

Dear Prof. Noemi Waight, 

 We are grateful for your contribution to our manuscript. We would like to thank your for approving our paper.

# Peer review: Reviewer response for version 2

**DOI:** 10.5256/f1000research.56745.r85859

**Published:** 2021-06-01

**Authors:** Deniz Sarıbaş

**Affiliations:** 1 Elementary Education Department, Istanbul Aydin University, Istanbul, Turkey

Thank you for sharing interview questions in the appendix. It gives much clearer idea about the analysis procedure. However, it would be easier to understand the classification of teachers' worldviews if you give deeper information about the categorisation process. For example, you may add a paragraph and/or table explaining categorisation procedure by giving a few examples of responses to the questions. A table illustrating a brief definition of each category and teachers' explanations that were included in these categories may be helpful.

 Chi-square analysis is a good choice to show the significance of the difference between three group of teachers. Thanks for adding this analysis.

Is the work clearly and accurately presented and does it cite the current literature?

Yes

If applicable, is the statistical analysis and its interpretation appropriate?

Not applicable

Are all the source data underlying the results available to ensure full reproducibility?

Partly

Is the study design appropriate and is the work technically sound?

Yes

Are the conclusions drawn adequately supported by the results?

Yes

Are sufficient details of methods and analysis provided to allow replication by others?

Partly

Reviewer Expertise:

Science education

I confirm that I have read this submission and believe that I have an appropriate level of expertise to confirm that it is of an acceptable scientific standard, however I have significant reservations, as outlined above.

# Response

**Published:** 2021-06-10

**Authors:** Dina Tsybulsky

**Affiliations:** Technion - Israel Institute of Technology, Israel

Dear Prof. Deniz Sarıbaş, 

 Thank you very much for reviewing Version 2 of our manuscript and offering valuable suggestions for its improvement.

*"However, it would be easier to understand the classification of teachers' worldviews if you give deeper information about the categorisation process. For example, you may add a paragraph and/or table explaining categorisation procedure by giving a few examples of responses to the questions. A table illustrating a brief definition of each category and teachers' explanations that were included in these categories may be helpful."*

 Following your suggestion, we have added Table 1 to explain the categorization procedure and present a brief definition of each category and illustrative example quotes by teachers in the categories (please see p. 3). We have renumbered the tables throughout the manuscript accordingly.

# Peer review: Reviewer response for version 1

**DOI:** 10.5256/f1000research.31054.r82750

**Published:** 2021-04-20

**Authors:** Deniz Sarıbaş

**Affiliations:** 1 Elementary Education Department, Istanbul Aydin University, Istanbul, Turkey

The researchers interpreted the results based on the frequencies of the use of pronouns and words without providing any statistical analysis. Therefore, it is hard to decide whether the comparisions between the participants in different categories (outside observer, circumspect participant and conscientious participants) are statistically correct.

 Furthermore, it is not clear how the researchers classified these participants into three categories. Athough they declared that they are forbidden to share recorded and/or transcribed interviews with us, they could have shared the questions. The researchers also need to mention the implications of this study. Overall, it is an interesting research by giving insights of different teacher worldviews in a digital society. However, it needs the minor aforementioned revisions.

Is the work clearly and accurately presented and does it cite the current literature?

Yes

If applicable, is the statistical analysis and its interpretation appropriate?

Not applicable

Are all the source data underlying the results available to ensure full reproducibility?

Partly

Is the study design appropriate and is the work technically sound?

Yes

Are the conclusions drawn adequately supported by the results?

Yes

Are sufficient details of methods and analysis provided to allow replication by others?

Partly

Reviewer Expertise:

Science education

I confirm that I have read this submission and believe that I have an appropriate level of expertise to confirm that it is of an acceptable scientific standard, however I have significant reservations, as outlined above.

# Response

**Published:** 2021-05-13

**Authors:** Dina Tsybulsky

**Affiliations:** Technion - Israel Institute of Technology, Israel

Dear Prof. Deniz Sarıbaş, 

 Thank you very much for reviewing our manuscript and offering valuable suggestions for its improvement. We really appreciate that you have invested time into our work in these challenging times.

*"The researchers interpreted the results based on the frequencies of the use of pronouns and words without providing any statistical analysis. Therefore, it is hard to decide whether the comparisions between the participants in different categories (outside observer, circumspect participant and conscientious participants) are statistically correct."*

 Thank you very much for this comment. Following your suggestion, we have checked for the statistical significance using the chi-square test (please see Results section).

**

*"Furthermore, it is not clear how the researchers classified these participants into three categories."*

 We have explained how the designation into the three categories occurred referring to the data sources that were used (please see p. 3).

*"Although they declared that they are forbidden to share recorded and/or transcribed interviews with us, they could have shared the questions."*

 Appendix 1 presents the interview questions ( Tsybulsky, 2021).

*"The researchers also need to mention the implications of this study."*

 On page 15, we have explained the study implications.

 Sincerely,

 Authors

# Peer review: Reviewer response for version 1

**DOI:** 10.5256/f1000research.31054.r78895

**Published:** 2021-03-02

**Authors:** Noemi Waight

**Affiliations:** 1 Department of Learning and Instruction, University at Buffalo, Buffalo, NY, USA

This manuscript focused on teachers’ discourse related to school digitization and understanding teachers’ worldviews in the educational-technology context. Specifically, this study explored science teachers’ worldviews in the educational-technological context by observing language behavior. While the focus on science teachers’ worldviews offered an alternative framework to examine teachers’ views, there were some important gaps in the narrative.

 Importantly, the manuscript attempted to map worldviews and language behavior, and while this was clearly articulated, what remained underdeveloped is the value of this connection for teaching and learning. In other words, it was not clear how this conceptualization was translated to inform practice. Here it would be important to engage the significance of this work and thus why the categorizations and linguistic patterns are important for science education.

 Based on my read, this manuscript was linked to a previous study that reported on worldviews and the categorization of teachers into three distinct groups. Yet, it was not clear how this designation occurred. What data sources were used to inform this categorization? The omission of this information is critical for understanding how linguistic patterns were related to the various types of teachers.

 Given the above, the notion of world views as a theoretical framework was limited and was only superficially addressed. This needs to be expanded.  

 As noted above, the organization of the findings based on the categorization of worldviews was a bit confusing since the manuscript indicated that this occurred in a different study. Yet, as a reader this was needed in order to understand how the data informed this categorization and the language behavior.

 The contributions of this work as reported in the manuscript requires more work. It was not clear how these contributions advance understanding of digitization in science teacher education? How did this work advance what we already know in this domain? In addition, I would suggest that the author(s) focus on the implications of their work for both practice and research.

Is the work clearly and accurately presented and does it cite the current literature?

Yes

If applicable, is the statistical analysis and its interpretation appropriate?

Not applicable

Are all the source data underlying the results available to ensure full reproducibility?

No source data required

Is the study design appropriate and is the work technically sound?

Yes

Are the conclusions drawn adequately supported by the results?

Yes

Are sufficient details of methods and analysis provided to allow replication by others?

Yes

Reviewer Expertise:

Science education; technology design and implementation; nature of technology; teachers' practices with technological-supported, inquiry-based practices.

I confirm that I have read this submission and believe that I have an appropriate level of expertise to confirm that it is of an acceptable scientific standard, however I have significant reservations, as outlined above.

# Response

**Published:** 2021-05-13

**Authors:** Dina Tsybulsky

**Affiliations:** Technion - Israel Institute of Technology, Israel

Dear Prof. Noemi Waight, 

 We are grateful for your valuable comments and suggestions that have contributed to improving our manuscript. The following are the detailed answers to your review.

*"Importantly, the manuscript attempted to map worldviews and language behavior, and while this was clearly articulated, what remained underdeveloped is the value of this connection for teaching and learning. In other words, it was not clear how this conceptualization was translated to inform practice. Here it would be important to engage the significance of this work and thus why the categorizations and linguistic patterns are important for science education."*

 Our study is not directly related to teaching and learning. However, since teachers are responsible for implementing the digitalization of today’s education by integrating ICT into education, their worldviews play a crucial role in this process (please see p.3). Our work contributes to understanding deep subconscious changes in teachers’ worldviews reflected in their language behaviour. Our linguistic analysis made it possible to delve beneath the surface of explicit statements to reveal implicit messages including their feelings, emotions, concerns, and attitudes. While content analysis (used in the previous study) deals with explicitly conveyed messages and views, the linguistic analysis performed in this study was aimed to provide a deeper understanding of the teachers’ worldviews based on implicit aspects related to the way a message is conveyed in terms of language use rather than to its content. Thus, we show the importance of linguistic analysis for examining teachers’ views and beliefs. On page 15, we have explained the study contribution.

*"Based on my read, this manuscript was linked to a previous study that reported on worldviews and the categorization of teachers into three distinct groups. Yet, it was not clear how this designation occurred. What data sources were used to inform this categorization? The omission of this information is critical for understanding how linguistic patterns were related to the various types of teachers."*

 We have explained how the designation into the three categories occurred referring to the data sources that were used (please see p. 3).

*"Given the above, the notion of world views as a theoretical framework was limited and was only superficially addressed. This needs to be expanded."*

 Thank you very much for this comment. We have expanded our theoretical framework regarding worldviews and added the definition of worldview construct by referring to the classic definition by Wilber (1995) (please see p. 3).

*"As noted above, the organization of the findings based on the categorization of worldviews was a bit confusing since the manuscript indicated that this occurred in a different study. Yet, as a reader this was needed in order to understand how the data informed this categorization and the language behavior."*

 Page 3 presents our explanation of the categorization of worldviews.

*"The contributions of this work as reported in the manuscript requires more work. It was not clear how these contributions advance understanding of digitization in science teacher education? How did this work advance what we already know in this domain? In addition, I would suggest that the author(s) focus on the implications of their work for both practice and research."*

 On page 15, we have explained the study contributions and implications.

 We thank you again for the effort you invested in improving our manuscript. We believe these efforts have resulted in a much stronger paper. It is very much appreciated.

 Sincerely,

 Authors
